# Mirror microbial life and the dual use dilemma

**DOI:** 10.1128/msphere.00342-26

**Published:** 2026-06-11

**Authors:** Michael J. Imperiale, Arturo Casadevall

**Affiliations:** 1 Department of Microbiology and Immunology, University of Michigan 242912, Ann Arbor, Michigan, USA; 2 Department of Molecular Microbiology and Immunology, Johns Hopkins Bloomberg School of Public Health 25802 , Baltimore, Maryland, USA; Virginia-Maryland College of Veterinary Medicine, Blacksburg, Virginia, USA

**Keywords:** mirror biology, dual use, biosecurity

## Abstract

Life as we know it on Earth contains sugars and amino acids that are, for the most part, in only one of two chiral forms. However, it has been proposed that molecules with the opposite chirality, called mirror molecules, could have beneficial uses. This possibility has led to discussions of producing replicating unicellular organisms with the opposite chirality, or mirror life. These concepts in mirror biology present potential dual use concerns. In this manuscript, we apply a framework we previously developed for considering the benefits and risks of gain-of-function experiments with infectious agents. We conclude that while mirror molecules are worth pursuing, the potential risks of mirror organisms currently far outweigh the benefits.

## PERSPECTIVE

In general, scientific research progresses at a steady pace, but it is occasionally vastly accelerated by the introduction of new technologies. In the life sciences, these technologies in recent decades have included recombinant DNA, DNA sequencing, PCR, and CRISPR, to name a few. All told, the benefits of this research have been enormous, enhancing human, animal, and plant life, as well as the environment. Each time a new technology is introduced, however, it is useful to pause and consider any potential harm that may ensue.

Most recently, mirror biology, encompassing research ranging from molecules to organisms, has entered the limelight. The potential benefits are extensive, from therapeutics and vaccines to disease surveillance and environmental detection. However, this technology presents new dual use considerations. In this essay, we will briefly describe how mirror biology offers both benefits and risks, applying the framework for dual use research that we originally developed regarding gain-of-function experiments (1).

## MIRROR BIOLOGY

Life on Earth has evolved such that the key informational molecules in the cell, proteins and nucleic acids, are produced from precursors that have chirality, or handedness. These are the l-amino acids and d-sugars, respectively. Scientists have had an interest in developing macromolecules with the opposite handedness, both as an intellectual pursuit and because of their potential applications. Currently, the highest level of interest appears to be in the production of peptides and proteins with d-amino acids. It is noteworthy that despite the overwhelming preference for l-amino acids in nature, some microbes use d-Ala and d-Glu in the construction of their cell walls, and some d-amino acids are used in eukaryotic peptides (2). d-Amino acids and peptides are more stable *in vivo* and in the environment, making them attractive candidates for applications such as therapeutics and environmental sensors, respectively. Currently, however, it is difficult to produce these peptides and proteins on a large scale. Unlike overexpression of recombinant l-chirality proteins in cells, natural ribosomes cannot incorporate d-amino acids into proteins: one would need a mirror ribosome to translate mRNA, which presents a related obstacle because synthesizing ribosomes themselves at scale is also problematic. Therefore, some scientists have proposed producing a self-replicating mirror cell that can be used as a biological factory for mirror protein production.

The development of mirror cells has been the subject of significant debate in recent months. A large group of scientists in the field published a paper calling for a ban on the development of mirror cells (3). Their most compelling argument was that, based on current knowledge, such cells would be non-immunogenic and resistant to predation, and as such pose an existential threat to other forms of life.

## OVERSIGHT AND ANALYSIS

Given the potential risks associated with mirror biology, the logical question that needs to be asked is, can we minimize any risks without unduly stifling important progress? This, of course, is the old “dual use dilemma” in the life sciences.

Since our service as inaugural members of the National Science Advisory Board for Biosecurity, we have devoted much thought to dual use research, especially as it applies to gain-of-function (GOF) experiments with pathogens that have pandemic potential (1, 4, 5). Of course, we are not the only people who have opined on this topic (6–8). Over time we witnessed many debates about GOF, synthetic biology, and related topics, and we realized that well-meaning and informed individuals on each side of this controversy could often not find agreement because the issues involved questions of risk and benefit, which are themselves questions of value that are not amenable to easy resolution. Consequently, we proposed a framework for considering these types of experiments that, in our opinion, today can also be applied to mirror biology (1, 5). Herein, we use this framework, which requires the following questions to be answered before performing an experiment that poses a significant threat of harm:

1. Does the research address a medical or scientific problem that needs to be solved to protect humanity from a significant threat?
2. Have the benefits and risks been carefully discussed and evaluated by a group of individuals with appropriate expertise (e.g., for mirror biology: infectious agents, biosafety and biosecurity, and public health)?
3. Does the research present inherent risks that can be minimized?

The answers to these questions depend on the aspect of mirror biology being studied. Certainly, we see no major concern in studying the natural roles of d-amino acids and l-sugars in biology. As noted above, there are ways in which mirror molecules such as peptides and proteins can be used as therapeutics against dangerous pathogens, including those with pandemic potential. In addition, these molecules can be incorporated into surveillance systems and used to detect potential threats in the environment. Furthermore, there are potential uses of l-sugars in cancer research (9) as well as non-calorie-producing sweeteners (10). Nonetheless, there is legitimate concern that if extremely stable d-proteins or l-sugars are used, any long-term detrimental effects of their presence in either a living organism or the environment are currently not known. Therefore, moving forward, it will be important to monitor for any adverse events. In sum, it appears that most mirror molecules largely meet the three criteria.

With respect to mirror organisms, as noted above, they could facilitate the production of these useful mirror proteins. On the other hand, given the argument that mirror organisms could theoretically pose an existential threat to humanity (3), it has been proposed that efforts be taken to improve other ways of producing d-proteins. This is similar to the argument championed by Lipsitch and Galvani for GOF experiments, in which they suggested that alternative, less dangerous approaches be used if they can answer the question more safely (11). We therefore posit that the first criterion has not been met for mirror organisms.

As for the second criterion, the benefits and risks of mirror organisms are being carefully discussed and evaluated. During the past year and a half, various venues, including the United Nations, brought together experts on biology and biosecurity to discuss mirror biology (12–16). To the best of our knowledge, the consensus of these discussions is that the risks of mirror organisms far outweigh any benefits.

Finally, it is unclear whether the risks of producing a mirror organism can be minimized at this time. One could argue that all work involving mirror organisms should be performed at the highest biocontainment level, BSL-4, but it seems that this is not practical if the intent is to produce large quantities of useful d-protein commercial products. Moreover, if the risk due to a lab escape is existential, then even the remotest possibility of that occurring must be taken seriously. We conclude, then, that the production of mirror organisms does not meet our criteria.

## OTHER CONSIDERATIONS AND CONCLUSION

Some bacterial capsules contain peptidoglycans composed of d-amino acids, and those molecules can stimulate antibody responses (17) and innate immunity (18). In fact, a conjugate vaccine using poly(γ-d-glutamic acid) elicited a T-independent antibody response against the *Bacillus anthracis* capsule and promoted IgG-mediated phagocytosis (19), indicating that at least the humoral arm of the adaptive immune system can recognize some mirror amino acids. However, it is likely that T-dependent responses cannot be generated due to the inability of the antigen processing machinery into cleave d-proteins to peptides for antigen presentation. Hence, the mammalian immune system is not completely defenseless against organisms expressing mirror life amino acids, but relying on T-independent responses for survival is likely not ideal.

In considering any potential threats posed by mirror life, it is worth entertaining whether such life already exists in the earth’s biosphere. The current consensus is that mirror life does not currently exist on Earth. The technical report that accompanies the paper by Adamala et al. (3) contains compelling arguments why this may be the case (20), but the fact is that we do not know for sure. Abiotic processes usually create racemic mixtures of chiral molecules used by life, including amino acids and sugars. If known life employing primarily l-amino acids and d-sugars emerged on Earth from a primordial soup of biomolecules, it is conceivable that other forms of life could have used molecules of different chirality, although it appears very unlikely that mirror life could evolve from known life. Hence, one can imagine life forms with d-amino acids and l-sugars that mirror extant biochemical processes. Life emerged relatively shortly after the Earth formed, and for most of the past 4.5 billion years, life was unicellular. Today, most of the microbial world remains unexplored, and it is estimated that 99% of microbes are unculturable with current microbiological techniques. Could some of these unculturable organisms fail to grow in standard microbiological media because they use opposite chirality molecules? The possibility of alternative microbial life on Earth has been considered, including noting that the methodology currently used for detecting microbial life could be inadequate to find such life (21). If mirror life or its variations already exist on Earth, then the known biosphere may be able to cope with them, and therefore the risk from mirror life may be less than if such life is alien to our biosphere. To be clear, we have no evidence that mirror life or its variations exist in the biosphere, but absence of evidence is not evidence for its absence, and perhaps the current debate on the safety of mirror life could stimulate an enhanced search for such life forms since their detection would be a tremendous scientific discovery of enormous consequences for our understanding of the origins of life and the versatility of biological forms.

It is reassuring to note that the discussions about the benefits and risks of mirror biology are happening well before any potentially dangerous experiments have been performed. In that sense, this debate differs from the debate over GOF experiments with dangerous pathogens, which was triggered by the production of mammalian transmissible highly pathogenic avian influenza viruses half a generation ago (22, 23).

We would also point out a similarity between the GOF and mirror biology debates. Specifically, both point to the need for clear terminology when discussing the issues. The term “gain of function” is often used loosely, without differentiating between what we would call everyday, standard GOF experiments that pose no risks and GOF experiments that could lead to the generation of a harmful agent. Inexact terminology has plagued the GOF debate, hindered understanding, and even led to heated arguments and accusations of perjury during congressional testimony. Similarly, discussions of mirror biology need to clearly differentiate experiments with single molecules from the production of a living organism.

In conclusion, in this analysis, we have applied a framework originally intended for a specific type of experiment, GOF, to another potentially risky area of biology. We think that the practical aspects of the criteria we developed—importance, open discussion, and safety—will continue to make them broadly useful to the scientific and policy communities.
